# Refining criteria for selecting candidates for a safe lopinavir/ritonavir or darunavir/ritonavir monotherapy in HIV-infected virologically suppressed patients

**DOI:** 10.1371/journal.pone.0171611

**Published:** 2017-02-13

**Authors:** Nicola Gianotti, Alessandro Cozzi-Lepri, Andrea Antinori, Antonella Castagna, Andrea De Luca, Benedetto Maurizio Celesia, Massimo Galli, Cristina Mussini, Carmela Pinnetti, Vincenzo Spagnuolo, Antonella d’Arminio Monforte, Francesca Ceccherini-Silberstein, Massimo Andreoni, On behalf of Icona Foundation Study and mono-PI/r database Study Cohorts

**Affiliations:** 1 San Raffaele Hospital, Milano, Italy; 2 University College London, London, United Kingdom; 3 National Institute for Infectious Diseases Lazzaro Spallanzani IRCCS, Roma, Italy; 4 Università Vita-Salute San Raffaele, Milano, Italy; 5 University of Siena, Siena, Italy; 6 University of Catania, Catania, Italy; 7 Luigi Sacco Hospital, Milano, Italy; 8 University of Modena, Modena, Italy; 9 San Paolo Hospital, Milano, Italy; 10 University of Rome Tor Vergata, Roma, Italy; San Antonio Military Medical Center, UNITED STATES

## Abstract

**Objective:**

The primary objective of this study was to estimate the incidence of treatment failure (TF) to protease inhibitor monotherapies (PI/r-MT) with lopinavir/ritonavir (LPV/r) or darunavir/ritonavir (DRV/r).

**Design:**

A multicenter cohort of HIV-infected patients with viral load (VL) ≤50 copies/mL, who underwent a switch from any triple combination therapy to PI/r-MT with either LPV/r or DRV/r.

**Methods:**

VL was assessed in each center according to local procedures. Residual viremia was defined by any HIV-RNA value detectable below 50 copies/mL by a Real-Time PCR method. Standard survival analysis was used to estimate the rate of TF (defined by virological failure or interruption of monotherapy or reintroduction of combination therapy). A multivariable Cox regression analysis with automatic stepwise procedures was used to identify factors independently associated with TF among nadir and baseline CD4+ counts, residual viremia, time spent with <50 HIV-RNA copies/mL before switch, history of virological failure, HCV co-infection, being on a PI/r and hemoglobin concentrations at baseline.

**Results:**

Six hundred ninety patients fulfilled the inclusion criteria and were included in this analysis. Their median follow-up was 20 (10–37) months. By month 36, TF occurred in 176 (30.2%; 95% CI:25.9–34.5) patients. Only CD4+ nadir counts (adjusted hazard ratio [aHR] = 2.03 [95% CI: 1.35, 3.07] for counts ≤100 vs. >100 cells/μL) and residual viremia (aHR = 1.48 [95% CI: 1.01–2.17] vs. undetectable VL) were independently associated to TF.

**Conclusions:**

Residual viremia and nadir CD4+ counts <100 cells/μL should be regarded as the main factors to be taken into account before considering switching to a PI/r-MT.

## Introduction

Ritonavir-boosted-PI based monotherapy (PI/r-MT) is considered by Italian guidelines a possible alternative switch strategy to standard combination antiretroviral therapy (cART) in case of drug toxicity [1]. Indeed, there is clear evidence that triple antiretroviral combinations are a cause of toxicities affecting different organs, such as kidney, bone, cardiovascular system. In most cases, nucleos(t)ide reverse transcriptase inhibitors (NRTIs) appear to be relevant drivers of these toxicities: exposure to abacavir (ABC) was associated with a higher risk of cardiovascular events [2–6], while the use of TDF was associated with potentially irreversible kidney damage [4, 7–13] and reduction in bone mineral density, with increased risk of fractures [14–17].

PI/r-MT has been tested in different randomized studies, showing that this switching strategy is safe in the large majority of subjects with undetectable viral load. These studies have also demonstrated that in case of failure, no PI-related resistance mutations were selected and re-introduction of triple therapy was successful, without loss of subsequent drug options [18–25].

The largest study conducted on PI/r-MT (PIVOT) showed that this strategy, with regular viral load monitoring and prompt reintroduction of combination treatment in case of viral rebound, preserved future treatment options and did not change overall clinical outcomes or frequency of toxic effects [23].

Different studies were able to identify a number of factors associated with failure to PI/r-MT, including nadir and baseline CD4+ count, duration of viral suppression, previous failure to ART, HCV co-infection, PI in the baseline cART, residual viremia levels at time of switch, hemoglobin levels, age, VL at cART initiation, gender, mode of HIV transmission [26–37]. In a previous study, we investigated factors associated to failure of LPV/r-MT and we found that factors associated with a lower risk of treatment failure (TF) were the duration of viral suppression <50 copies/mL prior to baseline and having LPV/r as part of last cART [38]. However, in that study the possible role of residual viremia in predicting failure of MT had not been investigated.

The primary objective of the current analysis was to estimate the incidence of virological and treatment failure of PI/r-based monotherapies with LPV/r or DRV/r in an unselected population with undetectable viral load achieved using a previous triple cART. Other objectives were to identify predictors of treatment failure in virologically suppressed patients undergoing simplification of cART with MT with PI/r and, based on the identified single predictors, to develop and refine a prediction score able to reliably anticipate failure to PI/r-MT.

## Methods

### Study population

This is a prospective study of a cohort of people who was followed-up prospectively at each of the clinical sites. The database for the analysis has been put together retrospectively using some specific criteria by including only patients who underwent a switch from any triple combination therapy to PI/r-based MT with either LPV/r or DRV/r and with a viral load suppressed to a level ≤50 copies/mL

Most of the included patients are currently under follow-up in the ICONA Foundation Study cohort (case-cohort nested within ICONA). ICONA Foundation Study (ICONA) is a multi-centre prospective observational study of HIV-1-infected patients, which was set up in 1997. Eligible patients are those starting cART when they are naive to antiretrovirals, regardless of the reason for which they had never been previously treated and of the stage of their disease. The ICONA Foundation study has been approved by IRB of all the participating centers; sensitive data from patients are seen only in aggregate form. All patients sign a consent form to participate in ICONA, in accordance with the ethical standards of the committee on human experimentation and the Helsinki Declaration (1983 revision). Demographic, clinical and laboratory data and information on therapy are collected for all participants and recorded using electronic data collection [www.icona.org]. Sites participating to the Icona Foundation Study could contribute by including additional patients (who formed the hereafter named “Mono PI/r database”) not enrolled in ICONA who satisfied the inclusion criteria for this protocol. Once included in the protocol, these additional participants have been followed-up using the same standardized monitoring procedures and data collection as that used for patients in the ICONA Foundation Study, until the date in which the database has been frozen for the analysis (July 31, 2015).

The main inclusion criteria were: i) having achieved a viral load ≤50 copies/mL while receiving triple combination therapy (cART) over follow-up with at least 2 consecutive measures below this threshold. The date of the second viral load was defined as baseline; the second condition in order to be included in this analysis was to have experienced a switch to monotherapy with LPV/r or DRV/r after baseline while current viral load was still ≤50 copies/mL. If a person had more than one episode of switch to one of the considered PI/r only the first ever occurring episode was included for this analysis. The date of switching after the first of two consecutive viral loads ≤50 copies/mL was defined as the baseline for this analysis.

Viral load was assessed in each center according to local procedures. A subset of individuals was tested by a Real-Time PCR method: in these cases, undetectable viremia could be defined in the presence of a “target not detected” result and residual viremia by any HIV-RNA value detectable below 50 copies/mL by a Real-Time PCR method. The reason why the remaining individuals were not tested by a Real-Time PCR method is that this assay was not available at the time of PI/r-MT start.

### Statistical analysis

Standard survival analysis was used to estimate the rate of virological failure (defined at the date of a confirmed [in two consecutive samples] viral load above a defined threshold). We used 50 copies/mL (main analysis) and 200 copies/mL (sensitivity analysis) as thresholds. A composite endpoint to define treatment failure (TF) was also used to study the durability of the monotherapy: in this analysis an event was defined by virological failure (defined as above), or by modification of monotherapy. TF was the primary outcome of this analysis.

Kaplan-Meier curves have been derived to estimate the time to event, with corresponding 95% confidence interval (CI), and thus to evaluate the durability of PI/r-MT. Unadjusted and adjusted relative hazards (RH) of the different endpoints have been calculated from fitting Cox regression models and tabulated.

Analyses were repeated after stratifying according to whether patients had been switched to a DRV/r- or LPV/r- based mono-therapy. A similar analysis has been performed to test for heterogeneity between the two sources of enrolment (“Icona patients” vs. other patients enrolled at Icona sites but not in Icona). Results of these additional analyses are available as supplemental online material only.

### Construction of the score

Besides the investigation of the predictive values of individual factors, we also aimed at constructing a score vector linear combination of these variables for prediction purposes. Before running the analyses, all factors previously identified as a predictor of mono PI/r-based therapy even in a single study (based on literature data) have been considered for inclusion in the score. These include the eight main candidates listed below:

- CD4 count at the date of switch (binary variable: ≤200 cells/μL vs. >200 cells/ μL)
- CD4 count nadir (binary variable: ≤100 cells/ μL vs. >100 cells/ μL)
- Duration of time with a VL ≤50 copies/mL before switching to monotherapy (continuous measurement, per 9 months longer)
-Evidence of previous virological failure on ART (categorical: No, yes to PI, yes to other drug classes)
- Co-infection with HCV (binary yes/no)
- Being on a PI/r-including regimen (binary yes/no) at the date of switch
- Haemoglobin (continuous measurement, per log_10_ higher)
- Viral load was fitted as two groups when it was assessed by a Real-Time PCR method: undetectable (target not detected) or residual viremia (i.e. any HIV-RNA detectable below 50 copies/mL by a Real-Time PCR method [Biomerieux NucliSENS EasyQ HIV-1 v.2.0, Siemens VERSANT HIV-1 RNA 1.5 Assay kPCR, Roche COBAS AmpliPrep/COBAS TaqMan HIV-1 Test or v.2.0, Abbott RealTime HIV-1]).

HCV-RNA was available only in a subset of individuals and was not used to classify people for HCV status.

Other important factors (e.g. resistance at baseline, HIV-DNA and adherence to treatment) could not be included as predictors, because they were not collected or were available only for a small subset of patients. We did however describe resistance (at baseline and/or at failure) in a subset of patients with available genotypic resistance testing (GRT) results. Baseline resistance data are result of historical tests performed at the time of previous virological failures using population sequencing in plasma. Patients with HIV-RNA >50 copies/mL confirmed in two consecutive samples or with HIV-RNA >200 copies/mL during follow-up were eligible for studying PI resistance by GRT.

The construction of the score followed the steps described below using a Cox regression analysis framework:

1. Best subset of the predictors out of the 8 a priori identified was chosen using automatic stepwise procedures (both 'backward' and ‘forward’ with Akaike information criterion and p = 0.05 as significance level and ‘best subset’ selection. The latter implies to fit all possible models including one, two, etc. up to all eight factors and identify the best fitting model using a chi-square for goodness of fit test).
2. The model which was consistently chosen by all three procedures was our final chosen best model. In addition, internal 5-fold cross validation in Icona (training set) and external validation (Mono PI/r database as validation) set was independently used to develop the score. This approach identified the same predictors selected by the steps described above (data not shown).

The Results of the full model including all eight factors have also been reported.

Cox regression prediction estimates of treatment failure for exact values of CD4+ count nadir were also calculated.

All analyses were performed using SAS version 9.4 (SAS Institute, Cary, NC, USA).

## Results

Six hundred ninety patients fulfilled inclusion criteria and were included in this analysis. Their median (Q1, Q3) age, nadir CD4+ count, current CD4+ count and duration of virological suppression below 50 copies/mL were 44 (37, 50) years, 359 (209, 633) cells/μL, 636 (482, 838) cells/μL and 44 (19, 75) months, respectively. Two hundred eight (30%) were female, 63 (9%) had a previous AIDS diagnosis, 95 (14%) previously failed to a PI, 537 (78%) were receiving a PI/r, 168 (24%) were co-infected with HCV and 323 (59%) out of 543 evaluable for residual viremia had undetectable viral load; further baseline characteristics are detailed in Table 1 and in S1 Table.

**Table 1:** Characteristics of patients starting PI/r-based monotherapy.

**Total number of patients studied**	690
**Female *n (%)***	208 (30.1%)
***Age*, *years*,** Median (IQR)	44 (37, 50)
***Mode of HIV Transmission*, *n (%)***	
IDU	164 (23.8%)
Homosexual contacts	203 (29.5%)
Heterosexual contacts	227 (32.9%)
Other/Unknown	94 (13.7%)
***AIDS diagnosis*, *n (%)***	63 (9.1%)
***HBsAg*, *n (%)***	
Negative	619 (89.7%)
Positive	0 (0.0%)
Not tested	71 (10.3%)
***HCVAb*, *n (%)***	
Negative	475 (68.8%)
Positive	168 (24.3%)
Not tested	47 (6.8%)
***Calendar year of baseline*,** Median (IQR)	2012 (2010, 2013)
***Baseline CD4+ count*, *cells/μL*,** Median (IQR)	636 (482, 838)
***<200 CD4+/μL*, *n (%)***	16 (2.3%)
***CD4+ count nadir*, *cells/μL*,** Median (IQR)	359 (209, 633)
***<100 CD4+/μL*, *n (%)***	74 (10.8%)
***Viral load at first cART*, *log***_***10***_ ***copies/mL*,** Median (IQR)	4.4 (3.3, 5.0)
***Site geographical position in Italy*, *n (%)***	
North	384 (55.7%)
Center	281 (40.7%)
South	25 (3.6%)
***Months from HIV diagnosis to date of switching to PI/r-monotherapy*,** Median (IQR)	149 (64, 230)
***Haemoglobin*, *g/dL*,** Median (IQR)	14.5 (13.3, 15.5)
***Duration of ART*, *months*,** Median (IQR)	72 (30, 149)
***Duration of VL suppression below 50 copies/mL*, *months*,** Median (IQR)	44 (19, 75)
***Previous failure to a drug class other than PI*, *n (%)***	170 (24.6%)
***Previous failure to a PI*, *n (%)***	95 (13.8%)
***PI/r in previous regimen*, *n (%)***	537 (77.8%)
***PI/r monotherapy with*, *n (%)***	
DRV/r	403 (58.4%)
LPV/r	287 (41.6%)
***VL at starting PI/r monotherapy*, *n (%)***	
Undetectable (Target not detected)	323 (46.8%)
Residual viremia	220 (31.9%)
Not classifiable	147 (21.3%)

**Fig 1:**
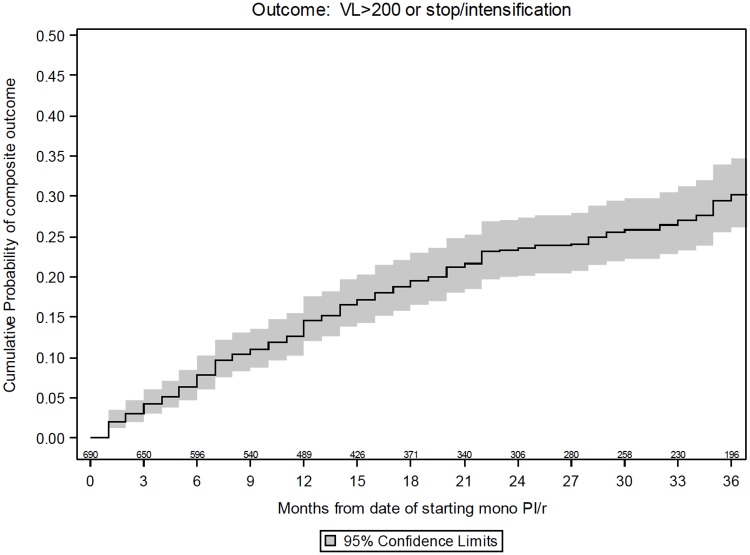
Kaplan-Meier estimates of time to HIV-RNA >200 copies/mL or stop or intensification of PI/r monotherapy.

The median follow-up was 20 (10, 37) months. By month 36, treatment failure occurred in 176 (30.2%; 95% CI:25.9–34.5) patients (Fig 1) with the following breakdown: 47 discontinuations (with the following regimen initiated not being reported), 105 intensifications (with or without interrupting the PI/r) and 24 pure confirmed virological failures >200 copies/mL. The reason for stopping were known for 33 of the 47 discontinuations. The main reasons were patient’s choice (n = 12, 32%), viral failure (n = 4, 11%), gastro-intestinal intolerance (n = 3, 8%) and simplification (n = 3, 8%). Viral load at time of starting a new drug was >50 copies/mL in 22 (21%) and >100 copies/ml in 14 (13%) of the intensifications.

**Fig 2:**
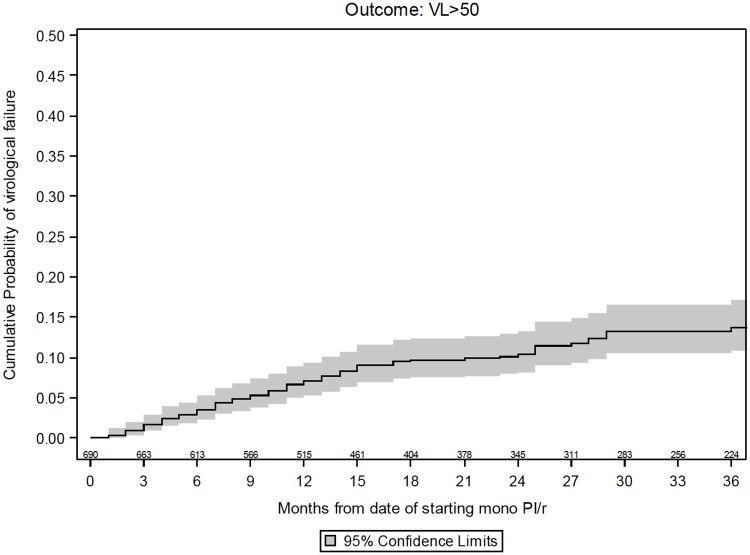
Kaplan-Meier estimates of virological failure >50 copies/mL.

**Fig 3:**
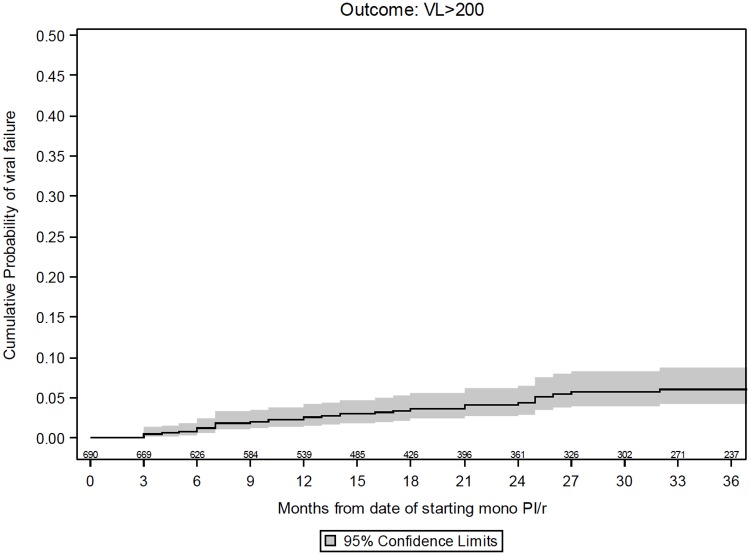
Kaplan-Meier estimates of virological failure >200 copies/mL.

The number of participants who experienced virological failure with viral load >50 and >200 copies/mL by 36 months was 71 (13.6% [10.6, 16.7]) and 31 (6.5% [4.2, 8.9]), respectively (Figs 2 and 3).

Table 2, S2 and S3 Tables illustrate the results of the univariate and multivariable analysis on the predictors of treatment failure: at the univariate analysis, factors associated with treatment failure were a nadir CD4+ count of ≤100 cells/μL vs. >100 cells/μL and the presence of baseline residual viremia vs. undetectable viral load (p<0.001 for both comparisons).

**Table 2:** Relative hazards of composite outcome from fitting a Cox regression analysis—PI/r-monotherapy score with all 8 pre-selected variables.

	Unadjusted and adjusted relative hazards of VL>200 or intensification			
	Unadjusted RH (95% CI)	p-value	Adjusted RH (95% CI)	p-value
**CD4+ count at starting PI/r monotherapy**				
*≤200 vs*. *>200 cell/μL*	2.34 (1.20, 4.59)	0.013	1.27 (0.55, 2.90)	0.573
**CD4+ count nadir**				
*≤100 vs*. *>100 cell/μL*	2.23 (1.55, 3.21)	< .001	2.03 (1.35, 3.07)	< .001
**Time with VL ≤50 copies/mL**				
*per 9 months longer*	1.20 (0.80, 1.80)	0.385	1.06 (0.68, 1.66)	0.787
**Previously failed virologically**				
*No*	1.00		1.00	
*Yes but not the PI class*	1.02 (0.69, 1.51)	0.924	0.97 (0.63, 1.51)	0.909
*PI class*	0.71 (0.44, 1.17)	0.182	0.70 (0.42, 1.18)	0.185
**HCV co-infection**				
*Yes vs*. *No*	0.93 (0.66, 1.31)	0.690	0.89 (0.61, 1.29)	0.522
*Not tested vs*. *No*	1.56 (0.91, 2.67)	0.108	1.63 (0.93, 2.83)	0.085
**On a PI/r-incuding regimen at starting PI/r monotherapy**				
*Yes vs*. *No*	0.95 (0.66, 1.36)	0.763	0.88 (0.59, 1.30)	0.512
**Haemoglobin**				
*per log*_*10*_ *higher*	0.16 (0.01, 2.27)	0.176	0.21 (0.01, 3.35)	0.268
**Viral load at starting PI/r monotherapy, copies/mL**				
*Undetectable (Target not detected)*	1.00		1.00	
*Residual viremia*	1.50 (1.05, 2.16)	0.028	1.48 (1.01, 2.17)	0.043
*Not classifiable*	1.83 (1.27, 2.64)	0.001	1.65 (1.10, 2.46)	0.015

Stepwise approaches removed all considered predictors (CD4+ count at starting PI/r-MT, time spent with viral load <50 copies/mL, history of virological failure, co-infection with HCV, being on a PI/r-including regimen at starting PI/r-MT, baseline hemoglobin level) but not nadir CD4+ cell count and residual viremia. This bivariable model was the best choice also when using the “best subset” selection, with adjusted relative hazards (RH) of 2.09 (95% CI: 1.06, 4.10, p = 0.03) comparing CD4+ nadir counts of ≤100 and >100 cells/μL and 1.75 (95% CI: 1.21–2.54, p = 0.003) comparing residual viremia vs. undetectable viral load.

**Fig 4:**
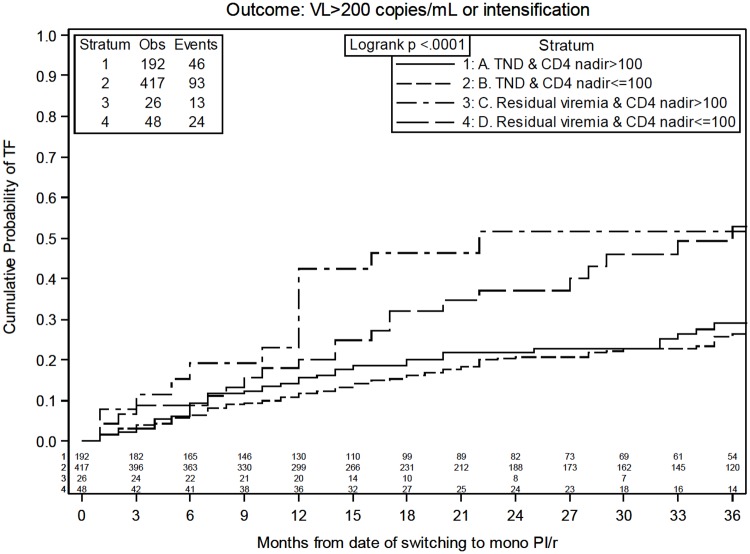
Kaplan-Meier estimates of time to HIV-RNA >200 copies/mL or stop or intensification of PI/r monotherapy according to main predictors strata. TND = target not detected.

Fig 4 shows Kaplan-Meier estimates of the composite outcome treatment failure after stratifying participants according to groups identified by the two main identified predictors. The 36-month estimated cumulative probability (95% CI) of treatment failure was 28.9% (20.8% - 37.0%) in the presence of baseline undetectable viral load and a CD4+ cells nadir of >100 cells/μL, 26.3% (21.0% - 31.6%) in the presence of baseline undetectable viral load and a CD4+ cells nadir of ≤100 cells/μL, 51.8% (31.8% - 71.8%) in the presence of baseline residual viremia and a CD4+ cells of >100 cells/μL, 52.9% (36.6% - 69.2%) with a baseline residual viremia and a CD4+ cells nadir of ≤100 cells/μL.

Table 3 shows the Cox regression prediction estimates of treatment failure for exact values of nadir CD4+ count, according to the presence or absence of baseline residual viremia.

**Table 3:** Cox regression prediction estimates of treatment failure for exact values of CD4 count nadir.

	Cox regression prediction estimates of treatment failure for exact values of CD4 count nadir	
	36 months predictions (%)	95% CI
***Group***		
**Undetectable VL**		
*CD4 nadir (cells/mm3)*		
50	40.0	31.4, 47.5
100	38.2	30.5, 45.0
200	34.7	28.5, 40.5
350	30.0	24.8, 34.8
500	25.8	20.7, 30.5
**Residual viremia**		
*CD4 nadir (cells/mm3)*		
50	43.9	32.8, 53.2
100	42.0	31.8, 50.7
200	38.3	29.5, 46.0
350	33.2	25.6, 40.0
500	28.6	21.4, 35.2

Results were similar when the analysis was repeated using the only Icona or the Mono PI/r database (data shown in supplementary tables only).

Table 4 shows available drug resistance data. In 17/54 (32%) patients, GRT was available both at baseline and at PI/r-MT failure and in other 13 (24%) patients GRT was available only at VF: in none of them newly selected PI-resistance mutations were selected at VF.

**Table 4:** Drug resistance mutations (DRMs) in patients who showed virological failure to boosted-protease inhibitors monotherapy and with genotypic resistance testing available at failure.

*Obs*	*DRMs at baseline*	*DRMs at failure*
*1*	**L10I**	**L10I**
*2*	**A71V**	**A71AV**
*3*	None detected	None detected
*4*	**L63P**	None detected
*5*	**L10I**	**L10I**
*6*	None detected	None detected
*7*	None detected	None detected
*8*	**M46I**	None detected
*9*	**L10V**	**L10V**
*10*	None detected	None detected
*11*	None detected	None detected
*12*	None detected	None detected
*13*	None detected	None detected
*14*	None detected	None detected
*15*	None detected	None detected
*16*	None detected	None detected
*17*	None detected	None detected
*18*	*Not done*	None detected
*19*	*Not done*	None detected
*20*	*Not done*	None detected
*21*	*Not done*	None detected
*22*	*Not done*	None detected
*23*	*Not done*	None detected
*24*	*Not done*	None detected
*25*	*Not done*	None detected
*26*	*Not done*	None detected
*27*	*Not done*	None detected
*28*	*Not done*	None detected
*29*	*Not done*	None detected
*30*	*Not done*	None detected

## Discussion

In the present study the number of participants who experienced virological failure by 36 months was between 6.5% and 13.6%, depending on the threshold used to define this event; by the same time interval from baseline, 30% experienced treatment failure, thus highlighting that in a relevant number of patients the PI/r-MT was interrupted because of toxicity or convenience. Our data do not allow us to analyze in depth the causes of MT discontinuation and thus to speculate further on this issue.

We identified in a large number of patients a nadir CD4+ count of ≤100 cells/μL and the detection of residual viremia (versus undetectable viral load) at the start of MT as the only predictors of failure to PI/r-MT. The results of this study suggest that these two predictors contribute both a fair amount of risk of failure although not in a synergistic manner (p-value for interaction = 0.88).

Although we were unable to define a true score for the outcome of PI/r-MT (as we identified only two predictors of response), we were able to provide robust independent estimates of failure for different nadir CD4+ counts, in the presence or in the absence of residual viremia at the time of switch to this strategy. Thus, our results are relevant as they document the prognosis of individuals treated with a strategy commonly used in clinical practice but with a limited support from available data. In addition, as estimates of failure are calculated using a large data set from a group of unselected individuals treated in every-day clinical practice, these estimates reflect more faithfully than those obtained from clinical trials the trends in the average HIV-infected individual in care in Italy. Finally, our results confirm those from previous studies, showing that PI-resistant HIV variants are almost never selected in patients experiencing virological failure.

In patients treated with LPV/r-MT, duration of previous viral suppression was a main predictor for long-term success of this strategy [26, 30, 38]; other important predictors of failure in one previous study were a CD4+ count nadir of <100/μL, a low hemoglobin level, a low adherence [27]; in a different study, failure to LPV/r-MT was associated with a CD4+ count nadir of <200 CD4+ [28] and a CD4+ count nadir of <200 was also a predictor of failure to a DRV/r-MT [37] and of PI/r-MT independent of the drug used [39]. However, in these studies residual viremia and nadir CD4+ counts never analyzed in the same multivariable model; our multivariable model combined all the previously identified risk factors for failure and identified only residual viremia and nadir CD4+ counts as those associated with failure. The results of our study also suggest that Hb is not a risk factor for failure to a PI/r-MT.

Beyond CD4+ nadir counts, recognized risk factors for failure in patients who received DRV/r-MT are the baseline presence of residual viremia, higher HIV-DNA load, shorter time of antiretroviral treatment before MT, as well as an adherence <100% during MT [26, 32–34, 36]. In the MONET Trial, HCV co-infection was independently associated to virological failure at week 48 [29]; however, considering week-144 results, and using the switches not considered failures endpoint, the only significant predictor of treatment failure was a baseline HIV RNA level > 5 copies/mL [35] and a meta-analysis of ten clinical trials has shown a significant higher risk of failure of any kind of ART in patients co-infected with HCV [31]. Our results suggest that HCV-coinfection is in fact not associated to failure to a PI/r-MT and confirm residual viremia as a key risk factor for failure.

HIV-DNA has been associated to a higher risk of failing MT [32, 33]. In a substudy of the MONET trial, HIV-1 DNA levels remained stable during 144 weeks of either DRV/r-MT or triple therapy with DRV/r + 2 NRTIs; furthermore, in both treatment arms, baseline HIV-1 DNA levels were predictive of plasma HIV-1 RNA detection during follow-up [34], although not clearly related to virological failure.

One limitation of our study is that we could not investigate baseline HIV-DNA load as a potential predictor of failure to a PI/r-MT, because this information was available for very few patients. Nevertheless, our finding that nadir CD4+ and the presence of baseline residual viremia predicts failure to PI/r-MT is in keeping with similar findings [27, 28, 32, 37] and suggest that the size of HIV reservoir is an important predictor of response to PI/r-MT; in fact, baseline HIV-1 DNA levels are predicted by the nadir CD4+ cell count [34, 40] and are strongly associated with residual viremia, independent of the ART history [41–44]. Therefore, our results also suggest that the nadir CD4+ counts and residual viremia are valuable proxies of HIV burden in reservoirs when considering switching to a PI/r-MT in a given patient. Moreover, it is worth noting that, although not specifically investigated in patients receiving PI/r-MT, in one study, residual viremia has been shown to be a better predictor of virological failure during ART than HIV-DNA [43].

The main limitation of this analysis is the observational context, as we cannot exclude that individuals undergoing MT have been selected as those with a better immune-virological response and a good tolerability to ART. Although, we tried to correct for all measurable confounders, we cannot exclude also that some unknown or unmeasured confounding still remained. A further limit is the absence of data on adherence; however, we believe that, under a clinical perspective, information on the role of nadir CD4+ cell count and residual viremia on the risk of failure to a PI/r-MT are truly important in selecting patients for this strategy, independent of adherence to therapy. As CSF samples were not prospectively collected we were not able to identify failures that may occur only in the CNS compartment. Finally, the follow-up of patients in the present study might have been too short to observe failures, in particular when virological failure occur at CNS, as recently highlighted by Kahlert and coll. [45].

In conclusion, in our large clinical setting, a PI/r-MT simplification strategy showed a risk of treatment failure consistent with that observed in clinical trials. Residual viremia and a CD4+ count nadir <100 cells/μL were the only predictors of failure to this strategy and should be thus considered the main factors to be taken into account before considering switching a virologically suppressed patient to a PI/r-MT.

## Supporting information

S1 Table Characteristics of patients starting PI/r-based monotherapy, according to cohort of enrollment. (DOCX)

S2 Table Relative hazards of composite outcome from fitting a Cox regression analysis—PI/r-monotherapy score with all 8 pre-selected variables (only patients from the ICONA Cohort). (DOCX)

S3 Table Relative hazards of composite outcome from fitting a Cox regression analysis—PI/r-monotherapy score with all 8 pre-selected variables (only patients from the Mono PI/r database). (DOCX)

S1 Dataset Raw data. (XLS)
