# Supplementary material for: Refining criteria for selecting candidates for a safe lopinavir/ritonavir or darunavir/ritonavir monotherapy in HIV-infected virologically suppressed patients
Source: PLoS One. 2017 Feb 13;12(2):e0171611. doi: 10.1371/journal.pone.0171611 (PMC5305227; doi:10.1371/journal.pone.0171611)
Supplement: S1 Table — (DOCX) [file pone.0171611.s001.docx]

| **Table 1S**. Characteristics of patients starting PI/r-based monotherapy, according to cohort of enrollment. | | | | |  |
| --- | --- | --- | --- | --- | --- |
|  | **Cohort of enrolment** | | | |  |
| **Characteristics** | **Icona** | **Mono-PI/r database** | **p-value^*^** | **Total** |  |
|  | N= 191 | N= 499 |  | N= 690 |  |
| ***Gender, n(%)*** |  |  | 0.007 |  |  |
| Female | 43 (22.5%) | 165 (33.1%) |  | 208 (30.1%) |  |
| ***Mode of HIV Transmission, n(%)*** |  |  | <.001 |  |  |
| IDU | 35 (18.3%) | 129 (26.0%) |  | 164 (23.8%) |  |
| Homosexual contacts | 82 (42.9%) | 121 (24.3%) |  | 203 (29.5%) |  |
| Heterosexual contacts | 66 (34.6%) | 161 (32.3%) |  | 227 (32.9%) |  |
| Other/Unknown | 8 (4.2%) | 86 (17.3%) |  | 94 (13.7%) |  |
| ***AIDS diagnosis, n(%)*** |  |  | <.001 | <.001 |  |
| Yes | 33 (17.3%) | 30 (6.0%) |  | 63 (9.1%) |  |
| ***CVD diagnosis, n(%)*** |  |  |  |  |  |
| Yes | 28 (14.7%) | 32 (6.4%) |  | 60 (8.7%) |  |
| ***HBsAg, n(%)*** |  |  | <.001 |  |  |
| Negative | 184 (96.3%) | 435 (87.2%) |  | 619 (89.7%) |  |
| Positive | 0 (0.0%) | 0 (0.0%) |  | 0 (0.0%) |  |
| Not tested | 7 (3.7%) | 64 (12.8%) |  | 71 (10.3%) |  |
| ***HCVAb, n(%)*** |  |  | <.001 |  |  |
| Negative | 158 (82.7%) | 317 (63.5%) |  | 475 (68.8%) |  |
| Positive | 29 (15.2%) | 139 (27.9%) |  | 168 (24.3%) |  |
| Not tested | 4 (2.1%) | 43 (8.6%) |  | 47 (6.8%) |  |
| ***Hepatitis co-infection, n(%)*** |  |  | <.001 |  |  |
| No | 154 (80.6%) | 290 (58.1%) |  | 444 (64.3%) |  |
| Yes | 29 (15.2%) | 139 (27.9%) |  | 168 (24.3%) |  |
| Not tested | 8 (4.2%) | 70 (14.0%) |  | 78 (11.3%) |  |
| ***Calendar year of baseline*** |  |  | <.001 |  |  |
| Median (IQR) | 2013 (2011, 2013) | 2011 (2010, 2013) |  | 2012 (2010, 2013) |  |
| ***Age, years*** |  |  | <.001 |  |  |
| Median (IQR) | 38 (33, 45) | 45 (39, 51) |  | 44 (37, 50) |  |
| ***CD4 count, n(%)*** |  |  |  |  |  |
| 0-200 cells/mmc | 5 (2.6%) | 11 (2.2%) | 0.767 | 16 (2.3%) |  |
| Median (IQR) | 644 (507, 889) | 635 (471, 820) | 0.096 | 636 (482, 838) |  |
| ***CD4 count nadir, n(%)*** |  |  |  |  |  |
| 0-100 cells/mmc | 27 (14.1%) | 47 (9.6%) | 0.084 | 74 (10.8%) |  |
| Median (IQR) | 270 (163, 374) | 462 (231, 690) | <.001 | 359 (209, 633) |  |
| ***Viral load at first cART, log10 copies/mL*** |  |  | 0.014 |  |  |
| Median (IQR) | 4.6 (3.6, 5.1) | 4.1 (3.0, 4.9) |  | 4.4 (3.3, 5.0) |  |
| ***Site geographical position, n(%)*** |  |  | <.001 |  |  |
| North | 67 (35.1%) | 317 (63.5%) |  | 384 (55.7%) |  |
| Center | 124 (64.9%) | 157 (31.5%) |  | 281 (40.7%) |  |
| South | 0 (0.0%) | 25 (5.0%) |  | 25 (3.6%) |  |
| ***Diabetes, n(%)*** |  |  | 0.869 |  |  |
| Yes | 3 (1.6%) | 7 (1.4%) |  | 10 (1.4%) |  |
| ***Total cholesterol, mg/dL*** |  |  | 0.023 |  |  |
| Median (IQR) | 190 (157, 214) | 195 (171, 223) |  | 193 (167, 221) |  |
| ***HDL cholesterol, mg/dL*** |  |  | 0.595 |  |  |
| Median (IQR) | 47 (38, 57) | 46 (39, 58) |  | 46 (39, 57) |  |
| ***Months from HIV diagnosis to date of switching to PI/r-mono*** |  |  | <.001 |  |  |
| Median (IQR) | 62 (38, 160) | 170 (93, 241) |  | 149 (64, 230) |  |
| ***Haemoglobin, g/dL*** |  |  | 0.226 |  |  |
| Median (IQR) | 14.6 (13.4, 15.5) | 14.4 (13.2, 15.4) |  | 14.5 (13.3, 15.5) |  |
| ***Duration of VL suppression below 50 copies/mL, months*** |  |  | 0.003 |  |  |
| Median (IQR) | 31 (18, 66) | 51 (20, 78) |  | 44 (19, 75) |  |
| ***Other drugs class previously failed, n(%)*** |  |  | 0.006 |  |  |
| Yes | 33 (17.3%) | 137 (27.5%) |  | 170 (24.6%) |  |
| ***PI previously failed, n(%)*** |  |  | 0.002 |  |  |
| Yes | 14 (7.3%) | 81 (16.2%) |  | 95 (13.8%) |  |
| ***PI/r in previous regimen, n(%)*** |  |  | 0.029 |  |  |
| Yes | 138 (72.3%) | 399 (80.0%) |  | 537 (77.8%) |  |
| ***Current PI/r, n(%)*** |  |  | <.001 |  |  |
| DRV/r | 144 (75.4%) | 259 (51.9%) |  | 403 (58.4%) |  |
| LPV/r | 47 (24.6%) | 240 (48.1%) |  | 287 (41.6%) |  |
| ***VL at starting mono PI/r, copies/mL, n(%)*** |  |  | <.001 |  |  |
| TND | 55 (28.8%) | 268 (53.7%) |  | 323 (46.8%) |  |
| Residual viremia | 97 (50.8%) | 123 (24.6%) |  | 220 (31.9%) |  |
| Not classifiable | 39 (20.4%) | 108 (21.6%) |  | 147 (21.3%) |  |
| ***Duration of ART, months*** |  |  | <.001 |  |  |
| Median (IQR) | 35 (22, 97) | 87 (39, 162) |  | 72 (30, 149) |  |
| ***Follow-up time, months*** |  |  | <.001 |  |  |
| Median (IQR) | 14 (7, 25) | 24 (11, 40) |  | 20 (10, 37) |  |
| ^*^Chi-square or Kruskal-Wallis test as appropriate | | | | |  |

By 36 months 52 (39.6%) of the 191 participants in Icona experienced treatment failure vs. 103 (27.2%) of the 499 in the mono PI/r database.
