# Supplementary material for: Refining criteria for selecting candidates for a safe lopinavir/ritonavir or darunavir/ritonavir monotherapy in HIV-infected virologically suppressed patients
Source: PLoS One. 2017 Feb 13;12(2):e0171611. doi: 10.1371/journal.pone.0171611 (PMC5305227; doi:10.1371/journal.pone.0171611)
Supplement: S2 Table — (DOCX) [file pone.0171611.s002.docx]

| **Table 2aS**. Relative hazards of composite outcome from fitting a Cox regression analysis – PI/r-monotherapy score with all 8 pre-selected variables **(only patients from the ICONA Cohort)**. | | | | |
| --- | --- | --- | --- | --- |
|  | **Unadjusted and adjusted relative hazards of VL>200 or intensification** | | | |
|  | **Unadjusted RH (95% CI)** | **p-value** | **Adjusted^*^ RH (95% CI)** | **p-value** |
| ***CD4 count at starting mono PI/r*** |  |  |  |  |
| <=200 vs. >200 cell/mm3 | 1.20 (0.29, 4.98) | 0.798 | 0.61 (0.13, 2.96) | 0.542 |
| ***CD4 count nadir*** |  |  |  |  |
| <=100 vs. >100 cell/mm3 | 2.20 (1.18, 4.11) | 0.014 | 2.16 (1.08, 4.32) | 0.030 |
| ***Time with VL<=50*** |  |  |  |  |
| per 9 months longer | 1.16 (0.46, 2.91) | 0.756 | 1.04 (0.38, 2.83) | 0.945 |
| ***Previously failed virologically*** |  |  |  |  |
| No | 1.00 |  | 1.00 |  |
| Yes but not the PI class | 1.14 (0.50, 2.60) | 0.764 | 0.64 (0.24, 1.65) | 0.353 |
| PI class | 0.92 (0.39, 2.18) | 0.855 | 0.80 (0.32, 1.97) | 0.626 |
| ***HCV co-infection*** |  |  |  |  |
| Yes vs. No | 1.62 (0.88, 2.99) | 0.121 | 1.47 (0.73, 2.98) | 0.282 |
| Not tested vs. No | 1.67 (0.23, 12.23) | 0.614 | 1.35 (0.18, 10.28) | 0.772 |
| ***Previous ART with PI/r*** |  |  |  |  |
| Yes vs. No | 1.37 (0.70, 2.65) | 0.358 | 1.09 (0.54, 2.21) | 0.814 |
| ***Haemoglobin*** |  |  |  |  |
| per log10 higher | 0.02 (0.00, 1.32) | 0.068 | 0.01 (0.00, 1.05) | 0.052 |
| ***Viral load at starting mono PI/r, copies/mL*** |  |  |  |  |
| TND | 1.00 |  | 1.00 |  |
| Residual viremia | 1.28 (0.58, 2.85) | 0.543 | 1.12 (0.49, 2.53) | 0.793 |
| Not classifiable | 2.78 (1.22, 6.33) | 0.015 | 2.62 (1.12, 6.13) | 0.027 |
