# Supplementary material for: Refining criteria for selecting candidates for a safe lopinavir/ritonavir or darunavir/ritonavir monotherapy in HIV-infected virologically suppressed patients
Source: PLoS One. 2017 Feb 13;12(2):e0171611. doi: 10.1371/journal.pone.0171611 (PMC5305227; doi:10.1371/journal.pone.0171611)
Supplement: S3 Table — (DOCX) [file pone.0171611.s003.docx]

| **Table 2bS.** Relative hazards of composite outcome from fitting a Cox regression analysis – PI/r-monotherapy score with all 8 pre-selected variables **(only patients from the Mono PI/r database).** | | | | |
| --- | --- | --- | --- | --- |
|  | **Unadjusted and adjusted relative hazards of VL>200 or intensification** | | | |
|  | **Unadjusted RH (95% CI)** | **p-value** | **Adjusted^*^ RH (95% CI)** | **p-value** |
| ***CD4 count at starting mono PI/r*** |  |  |  |  |
| <=200 vs. >200 cell/mm3 | 3.18 (1.48, 6.84) | 0.003 | 1.88 (0.71, 5.02) | 0.206 |
| ***CD4 count nadir*** |  |  |  |  |
| <=100 vs. >100 cell/mm3 | 2.19 (1.40, 3.43) | <.001 | 2.03 (1.19, 3.46) | 0.009 |
| ***Time with VL<=50*** |  |  |  |  |
| per 9 months longer | 1.31 (0.83, 2.06) | 0.252 | 1.18 (0.70, 1.97) | 0.534 |
| ***Previously failed virologically*** |  |  |  |  |
| No | 1.00 |  | 1.00 |  |
| Yes but not the PI class | 1.04 (0.66, 1.64) | 0.865 | 1.05 (0.63, 1.77) | 0.845 |
| PI class | 0.66 (0.36, 1.21) | 0.179 | 0.69 (0.37, 1.32) | 0.265 |
| ***HCV co-infection*** |  |  |  |  |
| Yes vs. No | 0.85 (0.56, 1.28) | 0.428 | 0.79 (0.50, 1.24) | 0.301 |
| Not tested vs. No | 1.75 (0.99, 3.10) | 0.056 | 1.80 (0.99, 3.26) | 0.052 |
| ***Previous ART with PI/r*** |  |  |  |  |
| Yes vs. No | 0.84 (0.54, 1.30) | 0.429 | 0.80 (0.50, 1.29) | 0.357 |
| ***Haemoglobin*** |  |  |  |  |
| per log10 higher | 0.35 (0.01, 11.20) | 0.555 | 0.55 (0.01, 20.96) | 0.747 |
| ***Viral load at starting mono PI/r, copies/mL*** |  |  |  |  |
| TND | 1.00 |  | 1.00 |  |
| Residual viremia | 1.49 (0.96, 2.32) | 0.075 | 1.49 (0.93, 2.39) | 0.099 |
| Not classifiable | 1.48 (0.96, 2.27) | 0.075 | 1.16 (0.70, 1.92) | 0.569 |
